# Maternal Separation Induces Long-Term Alterations in the Cardiac Oxytocin Receptor and Cystathionine *γ*-Lyase Expression in Mice

**DOI:** 10.1155/2020/4309605

**Published:** 2020-01-24

**Authors:** Daniela C. Wigger, Nicole Gröger, Alexandra Lesse, Sabrina Krause, Tamara Merz, Harald Gündel, Katharina Braun, Oscar McCook, Peter Radermacher, Jörg Bock, Christiane Waller

**Affiliations:** ^1^Department of Psychosomatic Medicine and Psychotherapy, University Hospital Ulm, Germany; ^2^Department of Zoology and Developmental Neurobiology, Institute of Biology, Otto von Guericke University, Magdeburg, Germany; ^3^Institute of Anesthesiological Pathophysiology and Process Engineering, University Medical School, Ulm, Germany; ^4^Center of Brain and Behavioral Science (CBBS), Otto von Guericke University, Magdeburg, Germany; ^5^PG Epigenetics and Structural Plasticity, Institute of Biology, Otto von Guericke University, Magdeburg, Germany; ^6^Department of Psychosomatic Medicine and Psychotherapy, Paracelsus Medical University, Nuremberg General Hospital, Germany

## Abstract

We recently showed that blunt chest trauma reduced the expression of the myocardial oxytocin receptor (Oxtr), which was further aggravated by genetic deletion of the H_2_S-producing enzyme cystathionine *γ*-lyase (CSE). Exogenous H_2_S supplementation restored myocardial Oxtr expression under these conditions. Early life stress (ELS) is a risk factor for cardiovascular disease by affecting vascular and heart structures. Therefore, we tested the hypotheses that (i) ELS affects cardiac Oxtr and CSE expressions and (ii) Oxtr and CSE expression patterns depend on the duration of stress exposure. Thus, two stress paradigms were compared: long- and short-term separation stress (LTSS and STSS, respectively). Cardiac Oxtr expression was differentially affected by the two stress paradigms with a significant reduction after LTSS and a significant increase after STSS. CSE expression, which was significantly reduced in *Oxtr^−/−^* knockout hearts, was downregulated and directly related to Oxtr expression in LTSS hearts (*r* = 0.657, *p* = 0.012). In contrast, CSE expression was not related to Oxtr upregulation in STSS. Plasma Oxt levels were not affected by either ELS paradigm. The coincidence of LTSS-induced reduction of cardiac Oxtr and reduced CSE expression may suggest a novel pathophysiological link between early life adversities and increased risk for the development of cardiovascular disorders in adulthood.

## 1. Introduction

Maternal separation (MS) is a robust and widely used animal model for inducing early life stress (ELS), a paradigm that is applied using different protocols, especially regarding the duration and time point of stress exposure [1, 2]. Depending on the type of ELS exposure, rodents showed adaptive responses resulting in resilience to stressors encountered later in life, ultimately resulting in psychological and physiological well-being [3, 4]. Our previous studies in mice revealed that “mild” short-term separation stress (STSS) induced by MS during postnatal days (PND) 14-16 resulted in reduced depressive-like behavior in adulthood [5] and epigenetically regulated activation of synaptic plasticity gene expression in the hippocampal formation, which was paralleled by an increase in dendritic complexity and number of excitatory spine synapses [6]. In contrast, we observed that “chronic” long-term separation stress (LTSS) induced by MS from PND 1 to PND 21 and subsequent social isolation increased depressive-like behavior in adult males with elevated hippocampal Oxtr gene expression upon an adult stress challenge [5, 7]. Another study demonstrated that intracerebroventricular Oxt injections protected against the development of ELS-induced depressive-like behaviors through modulation of hippocampal mitochondrial function and neuroinflammation [8].

It is well established that Oxt acts not only as a neuromodulator, released from hypothalamic neurons, regulating social-emotional behavior [9], e.g., mother-child relationship [10], but also as a peripheral hormone. Beyond promotion of parturition and lactation [11], Oxt also critically influences peripheral organ functions [12]. In particular, Oxt exerts cardioprotective effects via negative chronotropic and inotropic properties [13], release of nitric oxide [14], anti-inflammatory and antioxidative properties [15], and modulating glucose utilization [16]. Since ELS, such as childhood maltreatment, may provide a critical programing factor for the development of coronary artery disease (CAD), diabetes [17], and hypertension [18] at later life periods, it is tempting to speculate that these effects might at least in part be mediated by ELS-induced changes of Oxt function. This hypothesis is supported by studies in animal models showing that MS results in cardiac changes including cardiomyocyte hypertrophy as well as cardiac fibrosis [19]. More data is available showing that MS results in changes on the vascular level by misprogramming of resistance artery smooth muscles [20], increased vasoconstriction [21], and blood pressure [22, 23]. These alterations are induced by superoxide production and endothelial dysfunction [24], inflammation [25], and sensitizing of the renal and sympathetic systems [26].

Hydrogen sulfide (H_2_S) produced by vascular and cardiac activity of CSE [27] is another factor known to exert protective effects in the cardiovascular (CV) system by relaxation of vascular smooth muscles, thereby inducing vasodilation and reduction of blood pressure [28]. Interestingly, these effects are also reported for the Oxtr system [14]. Finally, both the H_2_S [29] and oxytocin [30] systems exert their protective effects via antioxidant properties. However, this data might only suggest a possible interaction between the cardiac H_2_S and Oxtr system in ELS. The H_2_S-releasing salt NaHS attenuated the ELS-induced colonic epithelial damage, oxidative stress, and inflammation [31]. We have recently shown that the slow-releasing H_2_S donor GYY4137 restored the myocardial Oxtr expression in mice lacking the H_2_S*-*generating enzyme cystathionine *γ*-lyase (CSE) that had undergone cigarette smoke exposure to induce chronic obstructive pulmonary disease (COPD) prior to blunt chest trauma [32]. Hence, we hypothesized that ELS affects cardiac Oxtr expression and that these changes are dependent on the dose or duration of stress exposure. To test these hypotheses, we measured circulating Oxt plasma levels and cardiac tissue Oxtr protein expression in two different ELS paradigms: “chronic” LTSS and “mild” STSS. These measurements were complemented by analysis of the cardiac CSE expression, guided by the hypothesis that CSE is linked to stress-induced Oxtr changes in the heart.

## 2. Material and Methods

### 2.1. Animal Models

#### 2.1.1. Housing Conditions

C57BL/6 mice were used for the present study. All experimental animals were bred in our animal facility and housed on a 12 h light-dark cycle with food and water provided ad libitum. During pregnancy, the home cages were cleaned once a week to minimize pregnancy stress. After delivery of the pups (day of birth = postnatal day, PND 0), the home cages were not cleaned for the first 16 PND to minimize stress for the mother and her pups. To prevent potential litter effects, a split litter design was used, and males from different litters were randomly assigned to the four different experimental groups, two different stress groups, and the respective control groups (see below). Animals for the respective experiments were derived from at least 7 litters per group, with the exception of the Oxt plasma concentration analysis in the LTSS animals. Litter size was between 6 and 8 animals with random distribution of male : female ratio; however, only litters with near-equal male : female ratio were used for experiments. All animals were handled in accordance with the German guidelines for the care and use of animals in laboratory research. The protocols were approved by the ethics committee of the government of the state of Saxony-Anhalt (§8 TSchG; AZ: 42502–2-1272).

### 2.2. “Chronic” Long-Term Separation Stress (LTSS)

#### 2.2.1. LTSS Paradigm

Pups of this group were exposed to daily MS from PND 1 to PND 21 by removing them from the home cage and individually placing them in isolation boxes (13 × 13 cm, covered with paper bedding) for 3 h each day (9:00–12:00), which allowed olfactory and auditory but no visual or body contact with their separated siblings. During the first week, the isolation boxes were placed in a humidified incubator at 32°C. The dam remained undisturbed in the home cage. Prior to the return of the pups, fresh nesting material was provided in order to distract the mother from “overmothering” her pups during reunion. After weaning on PND 21, the animals were housed individually until the time of the respective experiment.

#### 2.2.2. Control Animals (CON)

Animals of this control group lived undisturbed with their mother and littermates. After weaning on PND 21, they were group housed with a maximum of 6 same-sex individuals until the time of the respective experiment on PND 64. Each experiment was conducted with a parallel individual control group. The control group used was identically treated as described here [7].

### 2.3. “Mild” Short-Term Separation Stress (STSS)

#### 2.3.1. STSS Paradigm

Pups of this group were separated from their mother on PND 14-16 using the same separation conditions as described for the LTSS group (see above). After the last separation session on PND 16, the pups remained undisturbed until weaning on PND 21. On PND 21, the animals were reared in groups with a maximum of 6 individuals until the onset of the experiments on PND 64.

#### 2.3.2. Control Animals (CON)

Animals of the control group were treated and housed as described for the control animals of the LTSS experiment.

### 2.4. Homozygous and Heterozygous *Oxtr* Knockout

Male *Oxtr*^−/−^ and *Oxtr*^+/-^ knockout mice were maintained on a mixed 129 × C57BL/6J genetic background as described by Takayanagi et al. [33]. For immunohistochemical analyses, two animals were used, and for Western blot analyses, one animal was used.

### 2.5. Immunofluorescence

Control hearts were embedded in Tissue-Tek O.C.T.™. Cryosections (7 *μ*m) were acetone-fixed and immunolabeled with the following antibodies: rabbit anti-Oxtr (1 : 1000, Sigma-Aldrich, St. Louis), mouse anti-smooth muscle actin (SMA, 1 : 100, Dako, Agilent, Santa Clara), Alexa Fluor goat anti-rabbit 488 (1 : 1000, Invitrogen, Carlsbad), and Alexa Fluor goat anti-mouse 555 (1 : 1000, Invitrogen, Carlsbad). Images were captured with a Leica DMI6000B microscope and edited with ImageJ 1.46.

### 2.6. Immunoblotting

On PND 64, mice were sacrificed by decapitation. Hearts were dissected, perfused with PBS, and immediately frozen in liquid nitrogen. For the lysate fractioning, whole hearts were mechanically homogenized in RIPA buffer containing phenylmethanesulfonyl fluoride (PMSF). Total lysates were obtained by centrifugation for 15 min at 4°C with 1000 × g. The membrane fraction used for Oxtr/Gapdh Western blot analyses was gained by centrifugation of total lysates for 30 min at 4°C with 12,000 × g. In preliminary tests, this fraction showed the highest protein expression level. Protein concentrations were measured using BCA assay (Pierce BCA Protein Assay Kit, Thermo Fisher Scientific, Waltham). 50 *μ*g protein was separated in a 12% sodium dodecyl sulfate-polyacrylamide gel and blotted to a polyvinylidene fluoride (PVDF) membrane (Membrane Hybond-P, GE Healthcare, Chalfont St. Giles). Visualization was performed using a ChemiDoc MP imaging system (Bio-Rad Laboratories, Hercules). The following antibodies were used: rabbit anti-Oxtr (1 : 1000, Sigma-Aldrich, St. Louis), mouse anti-Gapdh (1 : 500, Thermo Scientific, Waltham), horseradish peroxidase (HRP) goat anti-mouse (1 : 10,000, Invitrogen, Carlsbad), and HRP swine anti-rabbit (1 : 1000, Dako, Agilent, Santa Clara). Optical densities of protein bands were determined using ImageJ 1.46. Oxtr was normalized to the housekeeping gene Gapdh.

### 2.7. Immunohistochemistry

Additional groups of LTSS and STSS and their respective control animals as well as *Oxtr^−/−^* knockout hearts were formalin-fixed and paraffin-embedded. 4 *μ*m sections were stained with the following antibodies: rabbit anti-Oxtr (1 : 50, Proteintech, Manchester), rabbit anti-CSE (1 : 1000, Proteintech, Manchester), and secondary alkaline phosphatase- (AP-) conjugated goat anti-rabbit IgG (1 : 25, Jackson ImmunoResearch Europe Ltd., Cambridgeshire). The chromogen used for the AP reaction was AP red (Dako REAL Detection System, Dako, Denmark). Images were captured with a 10x lens using a Zeiss Axio Scope A1 microscope, and four 800,000 *μ*m^2^ sections per animal were quantified with Zeiss AxioVision Rel. 4.9.1 image analysis software. Results are presented as densitometric sum red.

### 2.8. Plasma Collection and Oxt Plasma Determination

After decapitation, 500 *μ*l blood was collected and 10 *μ*l EDTA solution (0.8 mg/ml EDTA, Carl Roth, Karlsruhe) was added. Next, 7.5 *μ*l of aprotinin solution (10 mg/ml aprotinin, Sigma-Aldrich, St. Louis) was added to 500 *μ*l of EDTA blood and centrifuged at 4°C for 5 min at 1600 × g. The supernatant was stored at -80°C until measurement. Oxt plasma levels were determined via radioimmunoassay (RIA, RIAgnosis, Sinzing, Germany).

### 2.9. Statistics

The statistical analysis was processed using SPSS software packages (Version IBM SPSS Statistics 24). For quantitative analyses, data are presented as median and interquartile range, if not stated otherwise. Data were tested for normal distribution using a Kolmogorov-Smirnov test. Due to the small sample size, nonparametric tests were used. The Mann-Whitney *U* test was performed for group comparisons. Correlations between Oxtr and CSE expressions were calculated using the Spearman rho test. Results were defined as significant at *p* ≤ 0.05.

## 3. Results

### 3.1. Localization of Oxtr in Cardiac Tissue of *Oxtr^−/−^*, *Oxtr^+/-^* Knockout, and Wild-Type Mice

**Figure 1:**
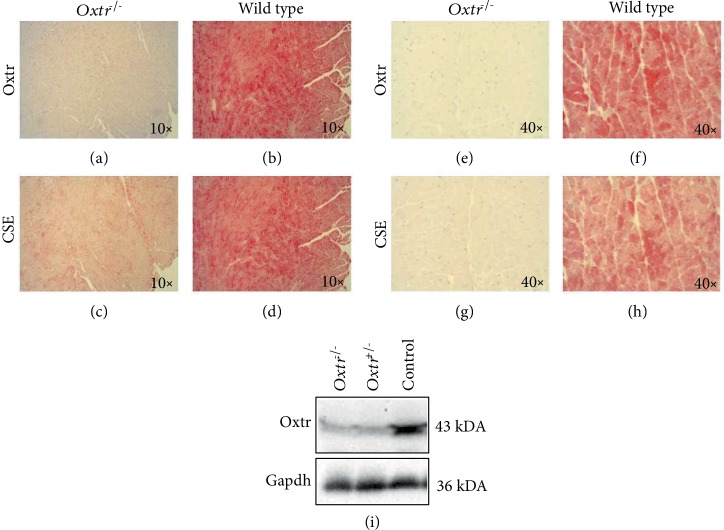
Immunohistochemical staining of Oxtr and CSE in the LV heart tissue in an *Oxtr^−/−^* knockout heart and a wild-type heart (×10 and ×40). Expression of Oxtr was absent in *Oxtr^−/−^* knockout heart (a, e) and clearly visible in wild-type myocardial tissue (b, f). CSE was significantly reduced in *Oxtr^−/−^* knockout hearts (c, g) compared to wild-type tissue (d, h). Western blot of *Oxtr^−/−^*, *Oxtr^+/-^* knockout, and control hearts confirming the specificity of the Oxtr antibody. Oxtr protein expression was substantially reduced in *Oxtr^−/−^* and *Oxtr^+/-^* knockout heart tissue compared to controls (i). LV: left ventricular; Oxtr: oxytocin receptor; CSE: cystathionine *γ*-lyase.

**Figure 2:**
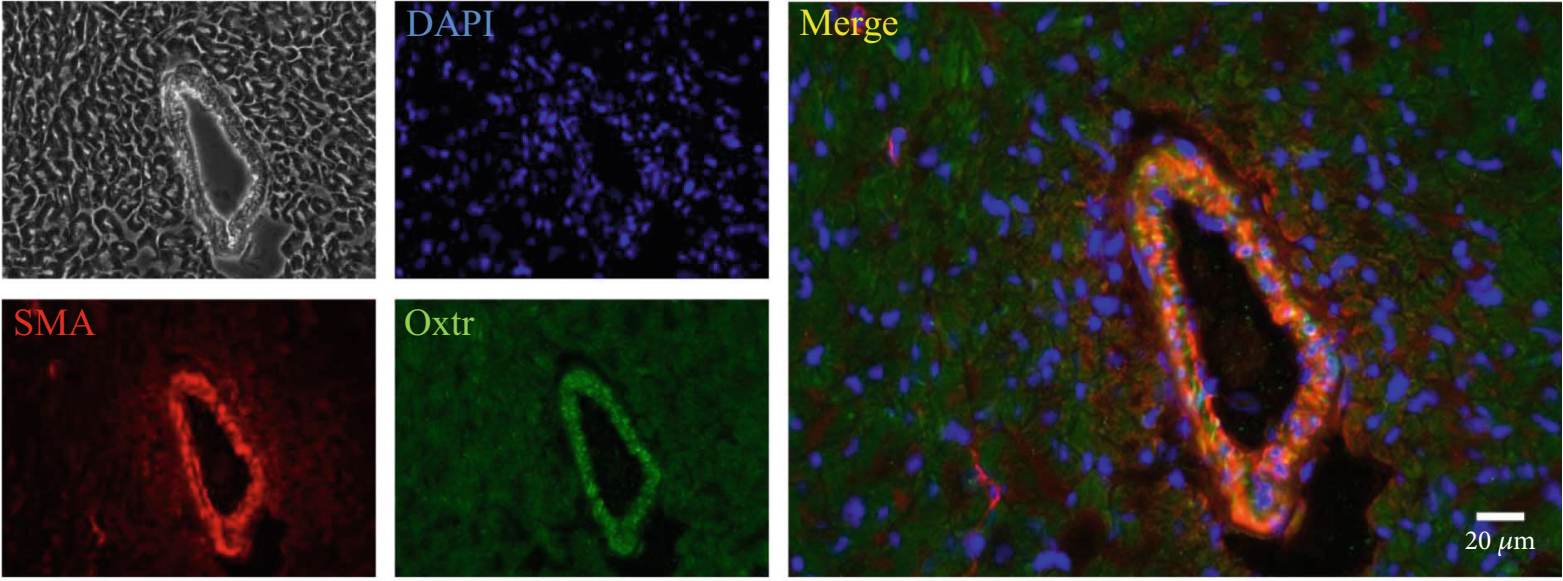
Immunofluorescence of the LV stained for Oxtr and SMA. Oxtr colocalized with SMA in a large arteriole indicating that Oxtr was highly expressed in arteriolar resistance vessels. LV: left ventricle; Oxtr: oxytocin receptor; SMA: smooth muscle actin.

Oxtr immunohistochemical staining was performed in left ventricular (LV) heart tissues (Figures 1(a), 1(b), 1(e), and 1(f)). Oxtr was visible in cardiomyocytes. The lack of Oxtr immunoreactivity in *Oxtr^−/−^* knockout mice and Western blot analyses in cardiac tissue of *Oxtr^−/−^*, *Oxtr^+/-^* knockout, and wild-type mice confirmed the specificity of our antibody. Oxtr protein was significantly downregulated in cardiac tissue of both *Oxtr^−/−^* and *Oxtr^+/-^* mice compared to wild-type mice (Figures 1(a), 1(b), 1(e), 1(f), and 1(i)). Immunofluorescence microscopy with costaining of Oxtr and smooth muscle actin (SMA) revealed that Oxtr is located in smooth muscle cells of the arterioles (Figure 2).

### 3.2. CSE Expression in LV Heart Tissue of *Oxtr^−/−^* Knockout and Wild-Type Mice

CSE staining in LV heart tissue in *Oxtr^−/−^* knockout mice showed a reduction of CSE protein compared to LV tissue of wild-type animals (Figures 1(c), 1(d), 1(g), and 1(h)).

### 3.3. Cardiac Oxtr and CSE Expression Is Downregulated after LTSS Exposure

**Figure 3:**
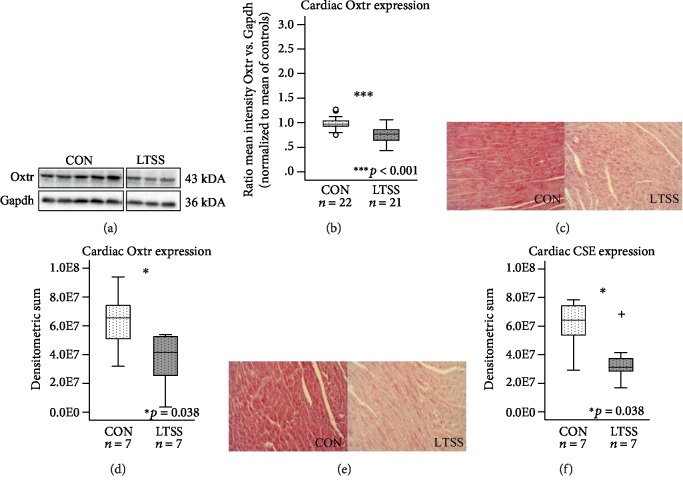
Western blot and immunohistochemical analyses of cardiac Oxtr and CSE expression in the LTSS paradigm. Representative Western blot of Oxtr protein expression (a) and quantitative results (b) revealed significantly reduced Oxtr expression in cardiac tissue of LTSS-exposed animals. Oxtr (d) and CSE (f) expression was significantly reduced in LTSS-exposed animals compared to their respective controls in immunohistological staining. Corresponding exemplary pictures of the LV myocardium are shown in (c, e). Data given as box plots (median, interquartile range, minimum, and maximum). ^+^Extreme value; Oxtr: oxytocin receptor; CSE: cystathionine *γ*-lyase; LTSS: long-term separation stress; CON: control; LV: left ventricular.

Quantitative Western blot analyses revealed a significant reduction of Oxtr protein expression in cardiac tissue of LTSS-exposed mice normalized to their unstressed controls (*p* < 0.001; Figures 3(a) and 3(b)). These results were corroborated by immunohistochemical stainings which confirmed the significant downregulation of Oxtr in cardiac tissue of LTSS-exposed animals compared to their controls (*p* = 0.038; Figures 3(c) and 3(d)). Immunohistochemistry revealed a significant downregulation of CSE protein in animals exposed to LTSS compared to their respective controls (*p* = 0.038; Figures 3(e) and 3(f)).

### 3.4. Cardiac Oxtr Expression Is Upregulated after STSS

**Figure 4:**
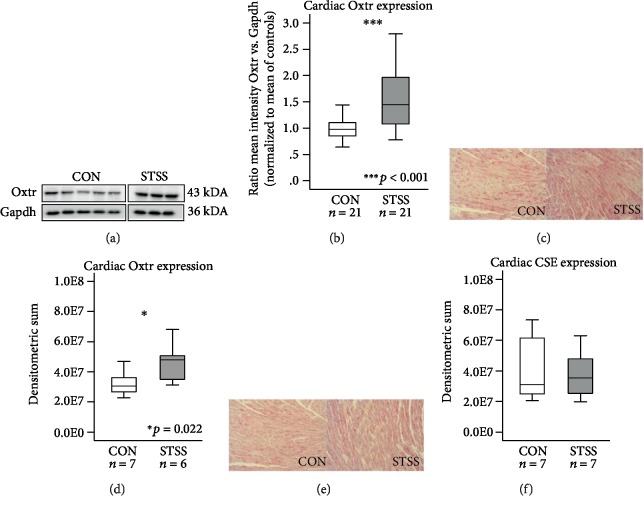
Western blot and immunohistochemical analyses of the cardiac Oxtr and CSE expression in the STSS paradigm. Representative Western blot of Oxtr protein expression in the STSS paradigm (a) and quantitative results (b) revealed significantly elevated Oxtr expression in STSS-exposed animals. Immunohistochemistry of Oxtr and CSE expression in the STSS-exposed animals compared to their respective controls revealed upregulated Oxtr expression after STSS (d). No significant changes in CSE expression were detectable after STSS (f). Corresponding exemplary pictures of LV myocardium are shown in (c, e). Data given as box plots (median, interquartile range, minimum, and maximum). Oxtr: oxytocin receptor; CSE: cystathionine *γ*-lyase; STSS: short-term separation stress; CON: control; LV: left ventricular.

Quantitative Western blot analyses revealed an upregulation of cardiac Oxtr protein expression after exposure to STSS normalized to controls (*p* < 0.001; Figures 4(a) and 4(b)). These results were in line with immunohistochemical stainings that confirmed elevated Oxtr in the heart of STSS-exposed mice compared to unstressed controls (*p* = 0.022; Figures 4(c) and 4(d)). Immunohistochemistry revealed that CSE protein expression remained unchanged in animals exposed to STSS compared to their respective controls (*p* = 0.710; Figures 4(e) and 4(f)).

### 3.5. Oxtr and CSE Interaction Effects in LTSS and STSS

**Figure 5:**
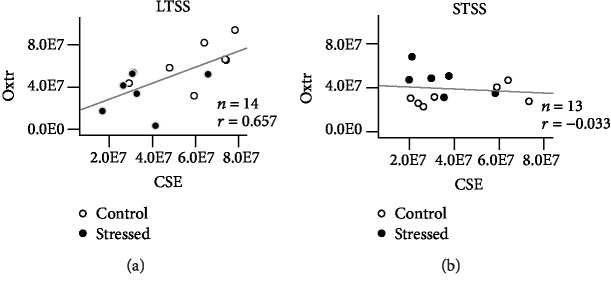
Regression analysis of Oxtr and CSE in the LV myocardium of LTSS- and STSS-exposed animals. Positive linear correlation between the Oxtr and CSE expression was detected in the LTSS paradigm (a). No significant correlation between Oxtr and CSE was found in the STSS paradigm (b). Oxtr: oxytocin receptor; CSE: cystathionine *γ*-lyase; LTSS: long-term separation stress; STSS: short-term separation stress; LV: left ventricular.

Correlation analyses between cardiac Oxtr and CSE expression yielded a linear correlation for LTSS and their respective controls (*r* = 0.657, *p* = 0.012; Figure 5(a)), whereas no statistically significant relation could be detected for STSS and their controls (*r* = −0.033, *p* = 0.553; Figure 5(b)).

### 3.6. Adult Oxt Plasma Levels Remain Unchanged after LTSS or STSS

No significant differences in the Oxt plasma concentrations were found between mice exposed to LTSS (5 pg/ml (4 pg/ml; 11 pg/ml); *n* = 4) and their respective controls (7 pg/ml (4 pg/ml; 16 pg/ml); *n* = 6; *p* = 1.0). In addition, no difference was found between mice exposed to STSS (62 pg/ml (45 pg/ml; 77 pg/ml); *n* = 18) and their respective controls (54 pg/ml (48 pg/ml; 89 pg/ml); *n* = 19; *p* = 0.964).

## 4. Discussion

The present study tested the hypotheses (i) whether two different ELS paradigms, i.e., “chronic” LTSS and “mild” STSS, affect the Oxtr and CSE expression in adult cardiac tissue, (ii) if this effect is “dose”-dependent, and (iii) whether ELS-induced changes of Oxtr and CSE expression are correlated. In line with these hypotheses, we found that early postnatal-induced changes of adult cardiac Oxtr expression were critically dependent on the “dose” of ELS exposure, while LTSS resulted in reduced Oxtr expression in adult cardiac tissue compared to controls; the opposite was found after STSS, i.e., upregulation of Oxtr expression. Finally, our study provides further evidence for an interaction between the CSE/H_2_S and oxytocinergic systems: in LTSS-exposed animals, reduced Oxtr expression was directly correlated with the CSE expression, which may indicate a functional interaction of these systems.

Recently, we showed in adult mice that combining cigarette smoke exposure with acute blunt chest trauma (physical trauma) markedly reduced cardiac Oxtr expression [32]. The present study revealed that neonatal chronic psychological trauma due to ELS exposure (LTSS paradigm) also leads to a long-term reduction in cardiac Oxtr expression. Interestingly, we found that these effects were dependent on the “stress-dose”: while “chronically” stressed LTSS animals displayed decreased Oxtr protein expression, “mild” STSS-exposed mice showed the opposite response, i.e., increased Oxtr expression. Applying the same stress paradigms, we previously demonstrated that LTSS increased depressive-like behavior in adulthood, an effect that was associated with alterations of the Oxtr expression in the hippocampus [7]. In contrast, STSS attenuated depressive-like behavior, paralleled by dendritic length, dendritic complexity, and spine number in the hippocampus, thus suggesting stress-induced adaptations of neuronal structures [5, 6]. Therefore, we conclude that “chronic” LTSS aggravates vulnerability, whereas “mild” STSS yields an opposite response by inducing adaptive processes that may promote resilience. In view of the CV protective functions of the Oxt/Oxtr system, it is tempting to speculate that LTSS-induced long-term reduction of cardiac oxytocinergic functions may confer a risk for developing CV dysfunctions. In fact, it was shown that low Oxtr expression in infarcted LV tissue coincided with aggravated myocardial injury [15] underlining that Oxtr reduction leads to adverse CV health, whereas elevated Oxtr expression induced by exposure to “mild” STSS might reflect cardioprotective effects. This hypothesis is supported by studies showing that increased Oxtr expression dampens atherosclerosis and protects against myocardial infarction [15, 34].

The lack of changes in Oxt plasma levels which we found in mice exposed to LTSS or STSS is in contrast to other studies reporting increased Oxt plasma levels after stress exposure in rodents [35, 36]. However, in these studies, Oxt plasma levels were measured 5 to 30 min after stress exposure whereas our analyses presented here were performed weeks after stress exposure at resting conditions. Due to its short circulating half-life [37], basal Oxt plasma levels might have normalized in adulthood after ELS exposure. In fact, our finding is in good agreement with our previous study in women with CM experiences, whose basal Oxt plasma levels were unchanged in adulthood [38], and another study also showing that Oxt plasma levels are independent of the dose of maltreatment [39]. Nevertheless, this does not exclude that ELS may exert a “programming” effect on acute Oxt release during stress challenges at later life periods.

The Oxtr is expressed in cardiomyocytes [40], in endothelial cells [41], and in the vascular wall of large vessels [42] where it exerts both negative ino- and chronotropic [13], antihypertrophic [43], and vasodilatatory effects, the latter via NO activation [41]. Our experiments presented here show that the Oxtr is expressed not only in smooth muscles of large vessels and isolated primary cells but also in smooth muscle cells of arterioles. Arterioles as the main regulators of vascular resistance contribute to blood pressure regulation via smooth muscle-mediated changes in vessel diameter. Follow-up analyses will assess if ELS induces additional changes in Oxtr expression directly in the arteriolar system.

We also showed reduced cardiac CSE expression in LTSS animals. Moreover, this reduction was positively correlated with the degree of Oxtr downregulation, which might suggest a direct interaction between the oxytocinergic and CSE/H_2_S systems in the heart, which is altered in response to LTSS. Similar to the Oxtr, CSE is also expressed in cardiomyocytes, endothelial and smooth muscle cells and, consequently, is involved in blood pressure regulation via H_2_S action [44]. Evidence for an interaction between Oxtr and CSE was provided in our previous study, which showed that *CSE^−/−^* knockout mice displayed a reduced cardiac Oxtr expression, which, in turn, was restored to the level of the control myocardial tissue by application of exogenous H_2_S [32]. In the present study, the reduced CSE expression observed in *Oxtr^−/−^* knockout mice further confirms a link between myocardial CSE and Oxtr expression. Moreover, the downregulation of Oxtr and CSE expression observed in the LTSS-exposed animals may contribute to CV pathology. In view of a possible reciprocal regulation between the Oxtr and CSE systems and the fact that mice lacking CSE show reduced endothelial-mediated vasorelaxation [45], this provides further evidence for a crucial role of Oxtr in blood pressure regulation. This view is further supported by the fact that Oxtr activation enhances baroreceptor sensitivity and, thus, enhances the capacity of blood pressure control [46, 47]. Finally, in ovariectomized spontaneously hypertensive rats, Oxtr blockade causes adverse cardiac remodeling [48] and monocrotaline-induced pulmonary hypertension leads to right ventricular Oxtr downregulation [49]. In contrast to the findings after LTSS, CSE expression was unchanged in the STSS animals. This suggest that any CSE-Oxtr interplay may be more relevant for vulnerable changes after LTSS but not for STSS, where, e.g., the dopaminergic system may be more involved [5].

## 5. Limitations of the Study

As methodological consideration, it should be pointed out that there was a substantial difference in the immunohistochemical CSE and Oxtr expression and in plasma Oxt concentrations in the two control groups for the two ELS paradigms. Since the two stress paradigms were run consecutively, two separate control cohorts were mandatory to obtain separate baseline data for each ELS paradigm. As previously shown [5], control animals from different cohorts may be divergent in basal parameters, as it is apparent in our study as well. Similar 10-20-fold variations of baseline plasma Oxt levels have been reported in a study on murine Oxt plasma levels using the same method as in the present experiment [50]. Furthermore, our data do not directly prove the causality between CSE and Oxtr since our experimental design allows only a first description of this relationship. Further studies will elucidate if both factors are pathophysiologically linked to each other.

## 6. Conclusion

Taken together, we show here for the first time that “chronic” ELS results in long-term reductions of myocardial Oxtr and CSE expression, which last until adulthood and which might be indicative of a biological interaction between the oxytocinergic and H_2_S systems. These alterations may reflect substantial biological pathways underlying microvascular and CV dysregulation in later life and may be viewed as “maltreatment” scar, i.e., a long-term negative outcome of ELS. The “chronic” LTSS stress paradigm provides a suitable model system, in which the cellular mechanisms underlying ELS-related CV disorders can be identified and characterized under experimentally controlled conditions.

## Data Availability

The data that support the findings of this study are available from the corresponding author on request.
